# Design of bile-based vesicles (BBVs) for hepatocytes specific delivery of Daclatasvir: Comparison of *ex-vivo* transenterocytic transport, *in-vitro* protein adsorption resistance and HepG2 cellular uptake of charged and β-sitosterol decorated vesicles

**DOI:** 10.1371/journal.pone.0219752

**Published:** 2019-07-16

**Authors:** Amira A. Boseila, Amal Y. Abdel-Reheem, Emad B. Basalious

**Affiliations:** 1 Department of Pharmaceutics, National Organization for Drug Control and Research, Cairo, Egypt; 2 Department of Pharmaceutics and Industrial Pharmacy, Faculty of Pharmacy, Cairo University, Cairo, Egypt; Laurentian, CANADA

## Abstract

Daclatasvir is a new direct acting antiviral used in treatment of Hepatitis C virus, in an attempt to increase its hepatocytes specificity and uptake. It was encapsulated within bile based vesicles (BBVs) containing egg phosphatidyl choline, cholesterol and sodium deoxycholate fabricated by thin-film hydration method. A D-optimal mixture design was applied to study the effect of formulation variables on vesicular characteristics. The dependent variables picked were the particle size, polydispersity index, zeta potential and entrapment efficiency. The optimized bile based vesicles were subjected for further modifications to prepare miniaturized anionic (ABBVs), cationic (CBBVs) and Sito-G decorated BBVs (Sito-GBBVs) to be capable to penetrate liver fenestrae (<200 nm). The aim of the current work is to compare the potential of the ABBVs, CBBVs and Sito-GBBVs loaded with Daclatasvir for stability in simulated biological fluids, ex-vivo intestinal transenterocytic transport, HepG2 cellular uptake and resistance to blood protein adsorption. The miniaturized ABBVs, CBBVs and Sito-GBBVs showed acceptable stability in simulated biological fluids. CBBVs had the highest transenterocytic transport through intestinal membrane. The internalization of CBBVs into HepG2 cells was about 2.1 folds that of ABBVs and 1.45 folds that of Sito-GBBVs. ABBVs and Sito-GBBVs showed superior resistance to opsonization compared to CBBVs which showed significant increase in particle size (p˃0.05) due to protein adsorption. The miniaturized Sito-GBBVs constitute a promising strategy to overcome key biological barriers facing hepatocytes specific delivery of Daclatasvir.

## Introduction

Hepatitis C virus (HCV) infection is the major cause for developing liver cirrhosis and hepatocellular carcinoma [1]. HCV infection is a global health issue that threats millions of people worldwide. Anti-HCV therapy currently aims to directly target HCV RNA and viral enzymes or influence host-virus interactions [2]. HCV is a plus-strand RNA virus encoding a single polyprotein that is proteolytically cleaved into 10 different parts [3], it's replication takes place in cytoplasmic membranous organelles [4]. It is stimulated by a concerted action of HCV replicase protein [5] together with host cell factors [6,7]. Recently it has been proclaimed that NS5A inhibitors block both RNA replication and assembly of HCV particles [8,9]. Drug under investigation in this study is Daclatasvir (DAC), a direct-acting antiviral agent that targets the nonstructural protein encoded by the NS5A protein of HCV [10].

Conventional vesicular nanocarriers systems showed a promising potential in improving the bioavailability of several therapeutic agents and immunogenic effect of biological therapeutics [11,12]. Unfortunately, nanocarriers suffer from several biological obstacles. First of all conventional vesicles have low stability in the GIT fluids which necessitated the improvement of their structures [13]. Different research work has exhibited the efficacy of incorporating bile salts into vesicular bilayer structure to improve their performance after oral administration [14,15]. It may be due to numerous factors including the defensive effect against GIT harsh conditions, membrane fluidizing capability and physicochemical properties of integrated bile [16,17]. The second biological barrier for nanocarriers to reach blood is their ability to pass through the gut epithelia. Nanocarriers must adhere to the mucus or possess facilitated diffusion through it leading to transenterocytic vesicular internalization [18]. The third biological barrier is blood protein adsorptions on nanocarriers' surface "opsonization" inducing their uptake by mononuclear phagocyte system (MPS) and excretion [19]. For effective delivery to hepatocytes, circulating vesicles should have smaller diameter than liver sinusoidal fenestrations (up to 150–200 nm), for ease extravasation into the space of Disse [20,21]. BBVs offer the gastrointestinal track stability required for the delivery of DAC to hepatocytes. However, the vesicles should be of small particle size and able to transport intact through the intestinal membrane into the portal circulation and resist to some extent the protein adsorption. Also, nanocarriers have to be capable for cell internalization through endocytic mechanisms [22]. The cellular uptake into hepatocytes is mediated through ligand endocytosis or membrane fusion. The ligand Sito-G was affirmed to be able to promote (Asialoglycoprotein receptor) ASGPR-mediated endocytosis. Also positively charged nanocarriers preferably conveyed to hepatocytes due to interaction with anionic group of ASGPR binding site followed by membrane fusion [23–26]. To the best of our knowledge, no endeavor has been accounted for comparing the capability of the anionic, cationic and Sito-G decorated BBVs to overcome the different biological barriers that encounter the delivery of drug into the liver cells. The optimized vesicles were tested for *in-vitro* biological fluids stability, *ex-vivo* transenterocytic vesicular internalization through the intestinal membrane and *in-vitro* cellular uptake using HepG2 cells as an *in-vitro* model.

## Materials and methods

### Materials

Daclatasvir (DAC) was kindly gifted by Marcyrl for pharmaceutical industries (Cairo, Egypt). β-sitosterol β-D-glucoside (Sito-G), Rhodamine B isothiocyanate (Rh B), stearylamine (SA), phosphate buffer saline (PBS) and dialysis membrane with 12000–14000 molecular weight cut-off were purchased from Sigma-Aldrich Chemical Co. (St Louis, MO, USA). Egg phosphatidyl choline 90% (EPC) was obtained from Fisher Chemical (UK). Cholesterol 95% was purchased from Acros Organics (New Jersey, USA). Sodium deoxycholate (SDC) was purchased from BASF Co. (Florham Park, New Jersey, USA). Triton X-100 was purchased from Fluka. Potassium dihydrogen orthophosphate, sodium hydroxide and Brij 35 were obtained from Merck (Darmstadt, Germany). Chloroform HPLC grade, Methanol HPLC grade, Acetonitrile HPLC grade and ethanol 75% were purchased from CARLO ERBA Reagents (France), Deionized water from Ultrapure (Type 1) water system (Direct–Q 3 UV) was used for the preparation of all buffer and water based solutions.

### Preparation of DAC-loaded BBVs

DAC-loaded BBVs were prepared by thin film hydration method [27,28]. DAC (5% w/w of total lipid weight), EPC, SDC and Cholesterol were dissolved in an organic solvent (Chloroform and Methanol in ratios 2:1 v/v). This organic solvent mixture was placed in round bottom flask and attached to a rotary evaporator (BUCHI, Rotavapor R-300, Germany) with a water bath adjusted at 60°C and reduced pressure 300 mbar, rotating at 120 rpm until the organic solvent evaporated completely leaving dried lipid thin film. The thin film was then hydrated by 10 ml deionized water for 1 h at 50°C and 120 rpm to obtain BBVs dispersion. The prepared BBVs dispersion was sonicated for 3 min in ultrasonic water bath (Crest ultrasonic, 575DAE, New York) to obtain vesicular dispersion then stored at 4°C until use.

A D-optimal mixture design model was constructed using Design expert software (version 10). The established independent variables, the responses and the formulation trials are illustrated in Table 1.

**Table 1:** Experimental runs, formulation variables, and measured responses of the D-optimal mixture experimental design.

BBVs	EPC (mg)	Chol (mg)	SDC (mg)	PS (nm)	PDI	ZP (mV)	EE%
**1**	185	15	0	788.35	0.72	-38.0	82.4
**2**	185	7.5	7.5	595.90	0.62	-43.0	94.7
**3**	170	30	0	906.90	0.63	-42.5	95.2
**4**	170	0	30	269.75	0.48	-45.9	93.5
**5**	155	30	15	514.95	0.53	-43.0	97.2
**6**	140	30	30	324.75	0.51	-39.2	91.7
**7**	170	30	0	662.75	0.43	-39.6	96.4
**8**	140	30	30	384.60	0.61	-43.5	88.7
**9**	155	15	30	336.50	0.50	-37.7	91.0
**10**	185	0	15	408.70	0.61	-41.8	97.0
**11**	170	15	15	601.45	0.52	-39.1	92.9
**12**	155	15	30	349.05	0.53	-44.8	88.5
**13**	200	0	0	439.15	0.79	-36.4	98.3
**14**	200	0	0	606.60	0.68	-37.3	89.9
**15**	170	0	30	348.05	0.58	-41.0	88.8
**16**	162.5	22.5	15	508.60	0.42	-41.4	88.2

All response values are represented as mean, (n = 3)

### Characterization of DAC-loaded BBVs

#### Measurement of particle size (PS), polydispersity index (PDI) and zeta potential (ZP)

The average PS and PDI of the prepared BBVs were determined by Zetasizer Nano ZS (Malvern Instruments, Malvern, UK) adopting the dynamic light-scattering mechanism. The ZP estimation was carried out in deionized water utilizing the same apparatus which observes the electrophoretic movability of the vesicles in an electrical field [29]. The dispersions were diluted in a ratio 1:20 with deionized water before measurement. All measurements were performed in triplicates.

#### Determination of DAC entrapment efficiency percent (EE %)

The prepared BBVs dispersion was centrifuged at 15000 rpm for 1.5 h at 4°C using cooling ultracentrifuge (Sigma 3K 30, Germany) [30]. The content of the unentrapped DAC in the supernatant was measured spectrophotometrically (Shimadzu, model UV-2450, Japan) at λ_max_ 316 nm. The EE of DAC was determined indirectly by subtracting the amount of unentrapped drug in the supernatant from the total amount of DAC added initially.

Drug EE% was calculated according to the following equation:

$$EE\%=\left(\frac{\left[Wt-Wf\right]}{Wt}\right)\times 100$$ (1)

Where, Wt symbolizes the total amount of the drug available in the formulation; Wf symbolizes the amount of the free drug exist in the supernatant [30].

#### Formulation optimization of DAC-loaded BBVs

In order to explore the impact of formulation variables on the characteristics of BBVs, DAC-loaded nanocarriers systems were prepared as indicated by the D-optimal mixture experimental design utilizing the Design-Expert software version 10 (Stat-Ease, Inc., Minneapolis, MN). Mixture design is usually applied when the formulation contains ingredients whose percentage sum is 100%. D-optimal design was chosen since it minifies the variance related to the estimates of the coefficients in the model. In this design, the amount of EPC (X_1_), the amount of cholesterol (X_2_) and the amount of SDC (X_3_) were picked as independent variables. The total concentration of the three variables was summed to 100%. The investigated responses were: particle size (PS; Y_1_), polydispersity index (PDI; Y_2_), zeta potential (ZP; Y_3_) and entrapment efficiency percent (EE%; Y_4_). The optimization procedures were aiming to accomplish PS (<500 nm), high EE% (>85%) and PDI (<0.6), and the value of ZP (<-25 mV).

#### Transmission electron microscopy (TEM)

The morphological features of the optimized DAC-loaded BBVs were visualized via TEM (JEOL, JEM-1230, Tokyo, Japan). One drop of the prepared dispersion was suitably diluted and adsorbed on a copper grid. The vesicles were negatively stained with an aqueous solution of phosphotungstic acid (2% w/v) for 5 min and then air dried at room temperature for 10 min before being inspected.

### Preparation of miniaturized charged BBVs and β-sitosterol decorated BBVs (Sito-GBBVs)

The optimized DAC-loaded BBVs were subjected to further modifications to improve their internalization and hepatocytes specificity. The particle size of the developed optimized BBVs (ABBVs) was further reduced by intense sonication of the dispersion of BBVs formed after hydration using an ultrasonic probe (VCX600, Sonics and Materials, Newtown, CT, USA) at power input of 100*W* for 3 min at 60 Amplitude. Miniaturized cationic BBVs (CBBVs) and β-sitosterol decorated BBVs (Sito-GBBVs) were prepared by incorporation of 5% w/w (of the total lipid weight) of stearylamine and Sito-G, respectively, into the organic solvent during the preparation of BBVs and using the same sonication power.

#### Determination of PS, PDI, ZP and EE

These characteristics were evaluated for ABBVs, CBBVs and Sito-GBBVs as previously mentioned.

#### Stability of the prepared BBVs in simulated biological fluids

Simulated gastric fluid (SGF) was prepared using a 34.2 mM NaCl solution in 50 ml deionized water at pH 1.2 adjusted with 1 M HCl. Pepsin (40 mg) was then added, followed by sodium taurocholate (2.15 mg) and phosphatidylcholine (0.76 mg) in 50 ml at 37°C [31]. Simulated intestinal fluid (SIF) was prepared by dissolving 6.8 g potassium dihydrogen orthophosphate in water and pH was adjusted to 6.8 with 1M NaOH then sodium deoxycholate (5 mM) was dissolved in the solution at 37°C [32].

BBVs dispersions (400 μl) were diluted up to 3.6 ml with SGF or SIF, and then incubated at 37°C for 1 h (SGF) and 4 h (SIF). The stability was assessed by estimating the effect on particle size distribution and percentage of DAC retained within BBVs formulations [32]. The quality characteristics of the prepared BBVs were compared to that of vesicular dispersion prepared with the same composition of the optimized BBVs without SDC (conventional liposomes).

$$DAC\%retained\;within\;vesicles=\frac{EE\%after\;incubation\;period}{EE\%before\;incubation\;period}\times 100$$ (2)

#### *In-vitro* release study

The *in-vitro* release study was adopted using a dialysis membrane of 12000–14000 molecular weights cut–off. Briefly, 1 ml of the different DAC loaded BBVs containing 1 mg drug, was placed in the dialysis bags tied from both sides with a thread. The receptor vessel was filled with 50 ml phosphate buffer pH 6.8 with 0.75% Brij 35 [33]. The receptor vessel was kept at 37±0.5°C in a shaker water bath (GLF Shaker 1038, Germany) with continuous stirring at 100 rpm. Samples of 1 ml were withdrawn at predetermined time intervals and resubstituted by the same volume of release media. The DAC content was determined spectrophotometrically at wavelength of 316 nm. The same procedures were performed for a suspension of DAC in deionized water containing 1 mg/ml drug to compare the *in-vitro* release of the developed BBVs with a conventional drug suspension.

### Determination of the transenterocytic internalization of BBVs

#### Animals

The aim of this study was to evaluate the capability of the ABBVs, CBBVs and Sito-GBBVs for transenterocytic internalization using non-everted gut sac technique. Experiments were approved by the Research Ethics Committee (REC; PT 1492) at Faculty of Pharmacy, Cairo University (Cairo, Egypt). Twelve male Wistar rats (200–250 g) were placed in a temperature and humidity-controlled room (23°C, 55% air humidity) with free access to water and standard rat chow. Rats were fasted overnight water access was permitted before the experiment. Rats were sacrificed by spinal dislocation. The small intestine was removed and washed cautiously with warm (37°C) 0.9% normal saline solution by a blunt tipped needle syringe, then intestinal pieces (8±0.2 cm long and 0.5±0.5 cm diameter) were ready [34].

#### Experimental protocol

DAC solution (in 0.1N HCL), the ABBVs, CBBVs and Sito-GBBVs (1 ml equivalent to 1 mg of DAC) were filled individually in an intestinal sac via micropipette 1000 μl, and the two sides of the intestine were tied firmly with a thread. Each intestinal sac was tied in the paddle rotating at 50 rpm in a mini-dissolution vessel containing 50 ml of 0.9% saline maintained at 37°C using USP II dissolution apparatus (Hanson Research, SR8PLUS, USA). Samples (1 ml) were withdrawn from the dissolution vessels at predetermined time intervals of 0.5, 1, 2, 4 and 8 h and replaced with same volume of fresh medium. The amount of DAC was determined in the samples both directly after withdrawal and after dilution of the withdrawn samples (five times with methanol) and sonication for 30 min. The assay of non-diluted samples represents the amount of drug permeated as free molecules. However, the assay of methanol-diluted samples represents the amount of drug permeated as free and encapsulated forms.

$$\%of\;transenterocytic\;internalization=\frac{Total\;drug\;permeated\;(free\;and\;encapsulted)-free\;drug\;permeated}{Total\;drug\;permeated}\times 100$$ (3)

#### HPLC determination of DAC

Samples were analyzed by an HPLC method developed using Agilent 1100 HPLC (Agilent Technologies, Santa Clara, CA, USA) instrument. Chromatographic separation of the samples was achieved by Zorbax SB, C18, 250 x 4.6 mm, 5 μm column (GL Sciences Inc., Torrance, CA, USA). The mobile phase consists of acetonitrile 30% and buffer 70% (composed of 0.05 M potassium dihydrogen orthophosphate adjusted to pH 3 with orthophosphoric acid). The mobile phase was pumped at flow rate 1 ml/min with UV detection at 310 nm. The injection volume was 20 μl and column temperature was kept at 30°C. A calibration curve was constructed within the concentration range of 0.8–20 μg/ml. The method has been validated for inter and intraday differences (the equation of linear regression for the calibration curve was y = 50.005x + 43.892; with coefficient R^2^ = 0.999, the calculated C.V. % values of interday and intraday precision were 0.33% and 0.56, respectively).

### *In-vitro* Cytotoxicity on HepG2 cells

HepG2 cells were seeded in Corning 96-well tissue culture plates at a density of (1×10^5^ cell*/*cm^2^). DAC solution, anionic, cationic and Sito-GBBVs were incubated with HepG2 cells for 24 h at different concentrations in serum-free media. After incubation period, the media was removed and replaced with 100 μl of neutral red saturated solution. The 96-well plates were then incubated at 37°C and 5% CO_2_ for 4 h. Then, media was cautiously removed and the cells were washed with 150 μl PBS per well followed by removing the washing solution by gentle tapping. Measure the absorbance of neutral red extract at 540 nm in a microtiter plate reader spectrophotometer, using blanks free from cells as a reference [35]. The 50% inhibitory concentration (IC_50_), the concentration required to cause toxic effects in 50% of intact cells, was evaluated by configuring a relation between viable cells and drug concentration.

### Cellular drug uptake assay on HepG2 cells

In cell culture flasks, HepG2 cell layers were initially treated with 0.25% trypsin/EDTA in DMEM (Dulbecco's Modified Eagle's medium), then the cells were re-suspended in the cell culture medium. Cells were then cultured in a 24 well plate at a cell density of 1×10^4^ cells/per well and incubated in a 5% CO_2_ incubator at 37°C for 24 h before incubation with drug formulations [36]. The cell association assay was carried out at 37°C in Opti-MEM containing DAC solution or tested DAC loaded BBVs preparations (10μg/ml) so that each well contained the same absolute amount of DAC. After 8 h, wells were washed with ice-cold PBS three times to cease incubation. The washed cells were lysed in PBS containing 0.5% Triton X-100, and then they were vortexed for 3 min. Then, the cell lysate was centrifuged to separate the cellular debris. The amount of DAC in the cell lysate was quantified by the validated HPLC previously mentioned.

### Confocal laser scanning microscopy imaging

The ABBVs, CBBVs and Sito-GBBVs were prepared using the same method used for preparing DAC-loaded BBVs; however, Rhodamine B (Rh B) in concentration of 0.5% of the total lipid substituted DAC in these preparations. Dialysis of the BBVs dispersions was done in deionized water using cellulose membrane (cutoff 12000) until clear dialyzed water was obtained indicating absence of free unentrapped Rh B. HepG2 cells were seeded on a glass bottom 10 compartments sterile tray. Then the cells were incubated with Rh B loaded BBVs for 2 hours at 37°C. After incubation period, the excess BBVs dispersions were removed by washing with cold PBS three successive times. Then 100 μl 75% ethanol (kept at -20°C) was used to fix the cells for 10 min and washed for three times with cold deionized water before imaging [37].

### Testing protein adsorption resistance of the prepared BBVs in serum

Protein adsorption resistance of DAC loaded BBVs was assessed by determining the alteration in the particle size following incubation of the BBVs in rat serum for 2 h [36]. Freshly prepared ABBVs, CBBVs, Sito-GBBVs and conventional liposomes were mixed with rat serum (50%, v/v in PBS) in a ratio of 1:1 (v/v) and incubated at 37±0.5°C. Dispersions were then checked for alteration in size after dilution by dynamic light scattering.

### Statistical analysis

The data acquired from different preparations were analyzed for statistical significance by one-way ANOVA using SPSS statistics program (version 21, SPSS Inc., Chicago, IL) followed by post hoc multiple comparisons. Differences were considered to be significant at p<0.05.

## Results and Discussion

### Formulation optimization of BBVs using D-optimal mixture design

In order to easily develop the optimal BBVs, D-optimal mixture experimental design was applied in the current study. The amount of EPC (X_1_), the amount of cholesterol (X_2_) and the amount of SDC (X_3_) were picked as independent variables. The investigated responses were: particle size (PS; Y_1_), polydispersity index (PDI; Y_2_), Zeta-potential (ZP; Y_3_) and entrapment efficiency percent (EE%; Y_4_). The responses of these formulations are summed up in Table 1. The independent and response variables were correlated using polynomial equation with statistical analysis through Design-Expert software. The values of the coefficients X_1_, X_2_ and X_3_ are related to the impact of these variables on the responses. A positive sign of coefficient indicates a synergistic impact while a negative sign indicates an antagonistic impact upon the response. The larger coefficient indicates that the independent variable has more powerful effect on the response [38].

### Investigation of preparation variables on the quality characteristics of DAC-loaded BBVs

#### Effect on particle size distribution and zeta potential of DAC-loaded BBVs

**Fig 1:**
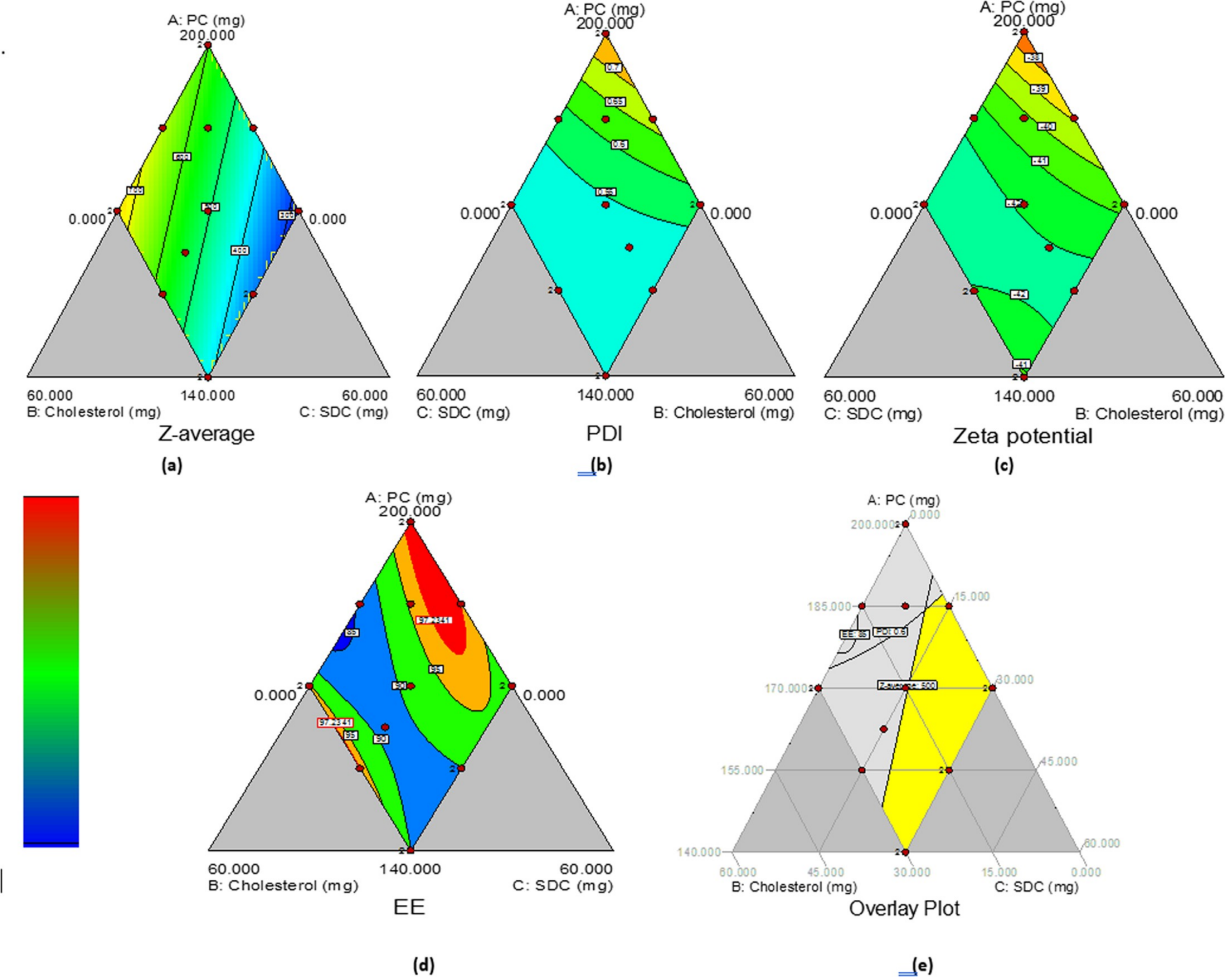
Contour plots of the impact of variables on the vesicle size (Y1) (a), PDI (Y2) (b), ZP (Y3) (c) and EE (Y4) of BBVs preparations and Overlay plot for the impact of different variables on the responses.

As illustrated in Table 1, the particle size of the different preparations of BBVs varied between 269.75±0.8 nm and 906.9±60.5 nm. It can be concluded that all preparation factors had an effective impact on the particle size. The approximation of response values of Y_1_ based on linear model was the most suitable because its PRESS was the smallest and the values of r^2^ and adjusted r^2^ were the highest. By applying ANOVA test, it can be interpreted that the terms X_1_, X_2_, and X_3_ had a significant impact on the PS (p<0.05). Fig 1A shows the contour diagram clarifying the impact of varying ratios of (X_1_), (X_2_) and (X_3_) on the PS of BBVs (Y_1_). The decrease in particle size as a role of SDC content in vesicles may be attributed to increased flexibility and lowered surface tension of the vesicles [14,27,28,39]. On the contrary, Cholesterol and EPC had a significant positive impact on particle size this could be ascribed to the increase in the lipophilicity and structure of the bilayer causing the development of stable vesicles consequently increasing the space required for hydrophobic drug encapsulation [40].

The PDI of the developed BBVs varied between 0.42±0.02 and 0.79±0.03 as shown in Table 1. The approximation of response values of Y_2_ based on reduced quadratic model was the most appropriate as its PRESS was the smallest and the values of r^2^ and adjusted r^2^ were the highest. ANOVA of the impact of variables on PDI of BBVs (Y_2_) outlined that all preparation variables had a significant impact on this response (p<0.05). The lower PDI indicates that the population of vesicles particle size is more homogeneously distributed [38]. The interaction term X_1_X_3_ has a significant impact on PDI (p = 0.0078). Fig 1B presents the contour diagram illustrating the impact of varying ratios of (X_1_), (X_2_) and (X_3_) on the PDI of BBVs (Y_2_). The lowest PDI values could be acquired at the intermediate and high values of SDC and also at the low and intermediate values of EPC.

The ZP of the developed BBVs varied between -36.4±1.7 and -44.8±1.3 mV. The approximation of response values of Y_3_ based on reduced quadratic model was the most adequate. ANOVA of the impact of variables on ZP of BBVs (Y_3_) showed that all preparation variables had a nonsignificant impact on this response (p>0.05). Fig 1C presents the contour diagram clarifying the impact of varying ratios of (X_1_), (X_2_) and (X_3_) on the ZP of BBVs (Y_3_). The vesicles’ surface charge affects the stability of the preparation. High positive or negative ZP values increase the repulsive forces which enhances the physical stability of the BBVs dispersion. The incorporation of EPC and SDC in the bilayer shell of the vesicles is responsible for imparting the high negative charge.

#### Effect on entrapment efficiency of DAC in BBVs

As illustrated in Table 1, the EE of the different preparations of BBVs varied between 82.4±2.4% and 98.2±3.2%. The approximation of response values of Y_4_ based on reduced cubic mixture model was the most adequate. ANOVA of the impact of variables on EE of BBVs (Y_4_) showed that all formulation variables and the interaction terms X_1_X_2_ and X_2_X_3_ had significant effects on this response (p<0.05). Fig 1D illustrates the contour diagram showing the impact of varying ratios of (X_1_), (X_2_) and (X_3_) on the EE of BBVs (Y_4_). The high values of EE (> 82%) for all preparations of DAC-loaded BBVs could be interpreted based on the characteristics of the drug and the preparation components. DAC is highly lipophilic, so it can be encapsulated easily in the bilayer of the phospholipids. EPC is a mixture of saturated and unsaturated acyl chains with a transition temperature below 0°C. The presence of the unsaturated phospholipids enhances the fluidity of the resulted bilayer and may provide more space for the solubilization of lipophilic drugs [41,42]. SDC could improve entrapment efficiency of DAC although this process is phosphatidyl choline dependent through increasing the flexibility of the bilayer, and improving the solubility of highly lipophilic agents in the bilayer [28,39]. Cholesterol enhances the incorporation of the lipophilic agents by increasing the hydrophobicity of the interfacial region and decreasing the permeability of the liposomal bilayer [42].

These results could be confirmed by the sign and values of the coefficient of independent variables in the regression equations for the given responses (Table 2).

**Table 2:** Regression results of the measured responses.

Response	Regression equation for the responses
$\frac{1}{\surd Y1}$	+ 0.000207899 X_1_ + 0.0000152440 X_2_ + 0.000711005 X_3_
**Y_2_**	0.003783 X_1_−0.00216 X_2_ + 0.035828 X_3_−0.00024 X_1_X_3_
**Y_3_**	-0.18539 X1−0.30673 X_2_ + 0.711107 X3−0.00643 X_1_X_3_
**Y_4_**	0.488968 X_1_ + 101.3865 X_2_−1.74636 X_3_−0.8005 X_1_X_2_ + 0.011836 X_1_X_3_−0.90781 X_2_X_3_ + 0.003811 X_1_X_2_X_3_ + 0.001476 X_1_X_2_(X_1_-X_2_)– 0.0021 X_2_X_3_(X_2_-X_3_)

#### Formulation optimization of DAC-loaded BBVs

The purpose of pharmaceutical preparations optimization is to define the variable levels from which a robust product with acceptable quality features can be fabricated. Some of the assessed responses should be kept at a minimum. In our study, these responses include the particle size (<500 nm) and PDI (<0.6). Some other responses, such as the EE (>85%) and ZP (<-25 mV) should be maximized to ensure reproducible upscaling and physical stability, respectively. The control methodology of the optimized BBVs was resolved to maintain procedure performance and output quality. The control space (or common used ranges) is set as the upper and lower limits for the preparation and procedure variables between which the parameters are routinely monitored during production so as to guarantee reproducibility. The control space should be within the design space. If the control space range is smaller than the design space, the procedure is then considered robust. In this case, the ideal operating ranges of the preparation variables (amount of EPC and the amount of SDC) for robust fabrication of BBVs are < 175 mg and > 20 mg, respectively (Fig 1E). BBVs loaded-DAC fulfilling these specifications were prepared and evaluated. An optimum response was found with Y_1_, Y_2_, Y_3_ and Y_4_ of 294.2±19.4 nm, 0.63±0.04, -37.7±0.6 mV and 92.4±2.4% at X_1_, X_2_ and X_3_ values of 160.54 mg, 16.8 mg and 22.64 mg, respectively.

### Morphological examination of the optimized DAC-loaded BBVs

**Fig 2:**
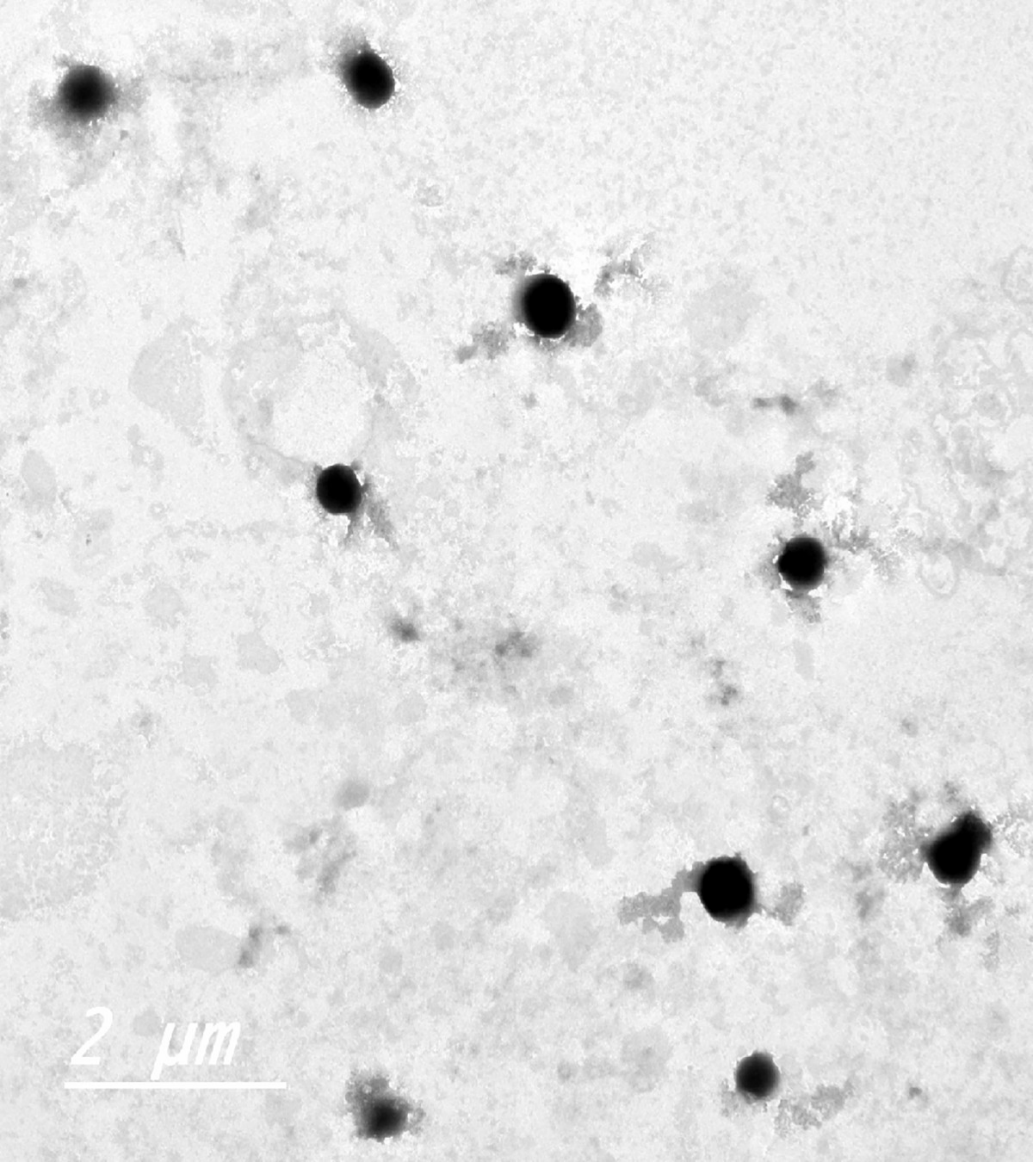
TEM micrograph of the optimized DAC-loaded BBVs.

Fig 2 shows TEM micrographs for the morphological examination of the optimized DAC-loaded BBVs. The photomicrographs demonstrated that all the prepared nanovesicles were distinct, non-agglomerated and round in shape. The vesicles size obtained from TEM ranged from 300–430 nm which is in agreement of the results obtained from PS determination.

### Characterization of miniaturized charged BBVs and β-sitosterol decorated BBVs (Sito-GBBVs)

The method of preparing BBVs was modified by subjecting the dispersion formed after hydration to sonication using ultrasonic probe for further miniaturization of BBVs below 200 nm to be able to penetrate the liver sinusoidal fenestrations. CBBVs and Sito-GBBVs were prepared by incorporation of stearylamine (positive charge inducer) and Sito-G, respectively into the miniaturized ABBVs to improve their internalization and specificity to hepatocytes. Nanocarriers systems, particularly liposomes, have been widely investigated for the targeted delivery of drugs to liver cells for the treatment of hepatic viral diseases [43,44].

Table 3 illustrates the quality characteristics of ABBVs, CBBVs and Sito-GBBVs compared to conventional liposomes. The vesicular size of the ABBVs, CBBVs, Sito-GBBVs and conventional liposomes were 145.45±4.88, 174.5±19.66, 165.4±28 and 168.3±11.6nm, respectively. The PDI of the different vesicular dispersions ranged from 0.352±0.007 to 0.535±0.05. The size of vesicles prepared under same conditions was affected by SA. Incorporation of SA and Sito-G in the lipid bilayers caused slight increase of PS [45,46]. The EE of the ABBVs, CBBVs, Sito-GBBVs and conventional liposomes were 92.6±2.3, 90.8±1.9, 91.6±2.7 and 89.6±1.5%, respectively. Thus, the incorporation of SA and Sito-G had no remarkable effect on entrapment efficiency of the developed BBVs. The ZP of the ABBVs, CBBVs, Sito-GBBVs and conventional liposomes were -37.7±4.2, +34.1±3.7, -39.7±4.1 and -22.3±2.1 mV, respectively.

**Table 3:** Effect of simulated biological fluids on different characteristics of prepared miniaturized BBVs.

	Initial	After incubation with SGF	After incubation with SIF
**Conventional liposomes** PS (nm) PDI % DAC retained			
	168.3±11.6	90.7±10.3	125.9±8.7
	0.352±0.007	0.9±0.1	0.8±0.09
	**-**	26.88±2.2	75.2±4.5
**ABBVs** PS (nm) PDI % DAC retained			
	145.45±4.8	117.6±7.3	129.9±10.04
	0.637±0.04	0.4±0.06	0.58±0.07
	**-**	72.9±3.8	92.2±4.1
**CBBVs** PS (nm) PDI % DAC retained			
	174.5±19.6	159.3±13.4	170.8±8.6
	0.561±0.02	0.45±0.03	0.52±0.03
	**-**	75.8±3.4	90.2±3.5
**Sito-GBBVs** PS (nm) PDI % DAC retained			
	165.4±28	149.8±9.7	162.4±13.3
	0.46±0.03	0.38±0.04	0.4±0.04
	**-**	76.44±2.5	90.8±3.7

All values are represented as mean ± SD, (n = 3)

### Stability of BBVs in simulated biological fluids

The effect of various biological fluids (SGF and SIF) on vesicular size and % of DAC retained, within the prepared BBVs, is illustrated in Table 3. The different BBVs containing SDC retained their vesicular size and PDI. On the other hand, SDC free liposomes showed significant reduction of vesicular size and an increase of PDI (p<0.05) indicating stability of bile based vesicles in GIT fluids compared to conventional liposomes. The stability imposed by BBVs was ascribed to the repulsion between the bile salts available in the vesicular membrane and the bile salts present in the gut [11]. Incubation in presence of SGF caused significant reduction (p<0.05) of the entrapped DAC in case of conventional liposomes compared to all BBVs. The basic nature of DAC could be the primary explanation behind the escape of the drug from the lipid bilayer of the vesicles especially the readily disrupted layer of the conventional liposomes.

### *In-vitro* release study

**Fig 3:**
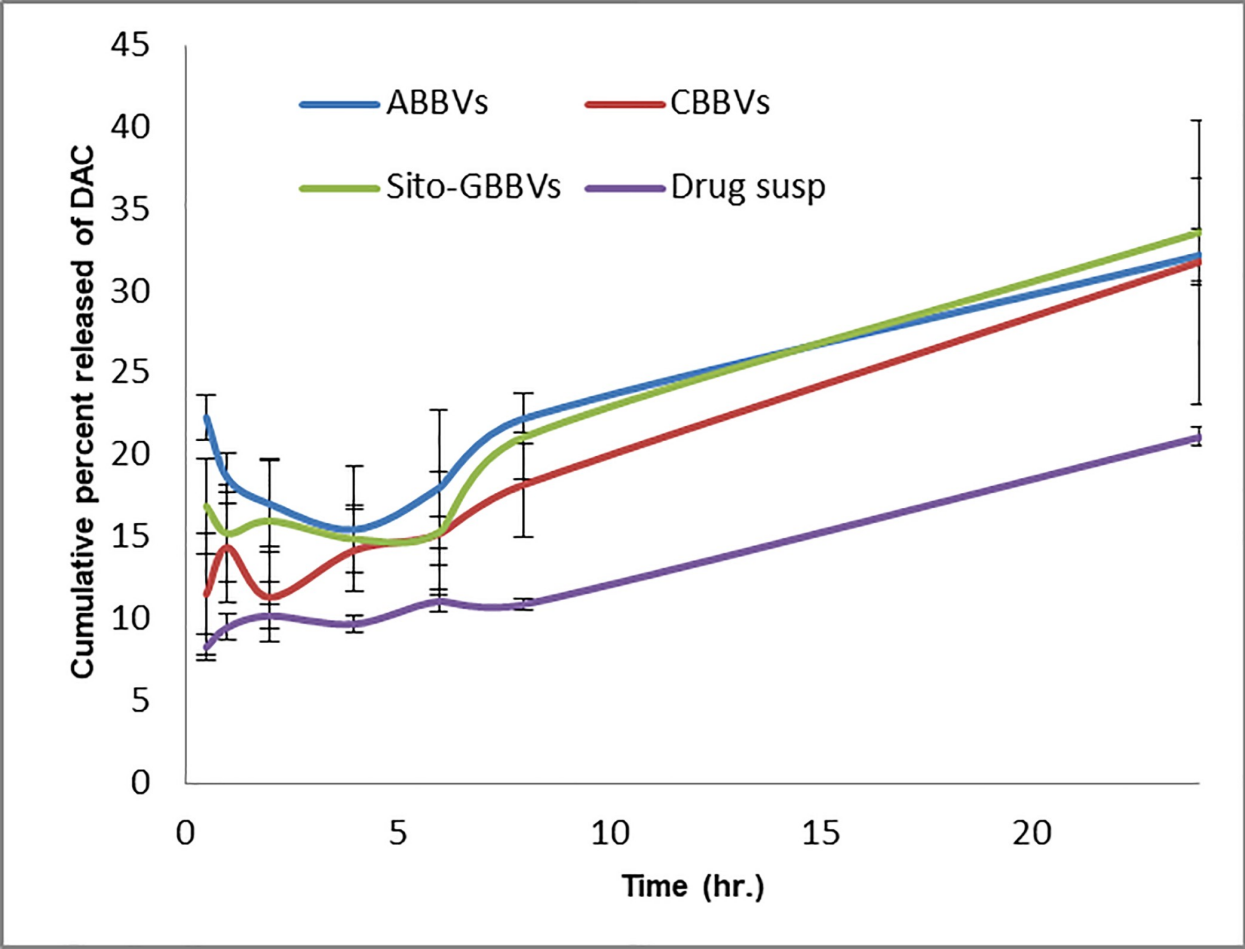
*In-vitro* release profiles of DAC from drug suspension, the ABBVs, CBBVs and Sito-GBBVs in phosphate buffer pH 6.8 with 0.75% Brij 35.

Fig 3 shows the *in-vitro* release profiles of DAC from prepared suspension, ABBVs, CBBVs and Sito-GBBVs in phosphate buffer pH 6.8 with 0.75% Brij 35. The release medium was suggested by FDA dissolution database for the dissolution of DAC tablets as this medium satisfies the sink condition required for the *in-vitro* release study [33]. The different preparations of BBVs showed higher release rate (ranged from 18.2±3.2 to 22.2±1.6% after 8 h) compared to drug suspension which showed 10.90±0.3% released after 8 h. The high release of DAC from the vesicular systems could be due to the presence of EPC and SDC which are surface active materials increasing the solubility and penetration of the free drug. The major disadvantages of the *in-vitro* dialysis method are the lack of complete mimicking of *in-vivo* digestion and neglecting the chance of vesicular disruption in physiological conditions [11].

### The transenterocytic vesicular delivery of BBVs

The transport of drug from vesicular dispersion through intestinal membrane could be done in the form of free drug or as the intact vesicles. Several authors have assumed the ability of entire BBVs to permeate through the stagnant water layer followed by uptake of the vesicles by membranous epithelial cells (M-cells) in the Payer’s patch [11] and through the transenterocytic transport and internalization of the vesicles [15]. For effective delivery to hepatocytes after administration, the intact vesicles should transport the intestinal membrane reaching the portal circulation and the liver cells. Then, these vesicles are taken up by liver cells through endocytosis followed by entry into the cytoplasmic matrix via the endosomal or lysosomal membranes [44]. Thus, the first barrier which faces the delivery of drug after oral administration is the absorption of intact vesicles into the blood. The intestinal permeation of the different BBVs has been compared to that of DAC solution for the selection of the vesicular system having the highest transenterocytic internalization. The permeation study was performed through using non-everted rat intestine in 0.9% saline. Non-everted intestinal sac had numerous advantages including simple preparation; small drug amount is needed, less intestinal morphological destruction, and capability for successive sampling from serosal compartment [34,47].

**Fig 4:**
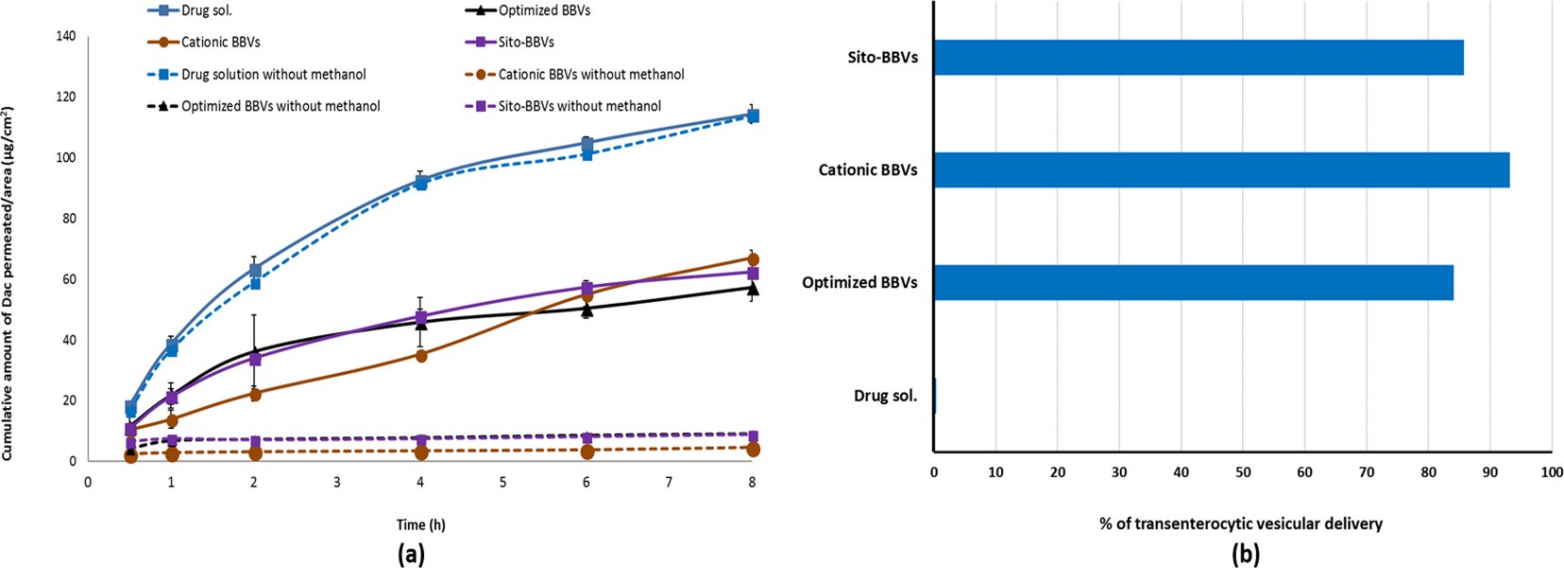
**(a) Cumulative amount permeated per unit area of DAC from ABBVs, CBBVs, Sito-GBBVs and drug solution through non-everted rat intestine in 0.9% saline at 37°C.** Data presented as mean ±SD (n = 3). Dotted line: before dilution with methanol. Solid line: after dilution with methanol. **(b) Percent of transenterocytic BBVs delivered through intestinal rat epithelial cells**.

Fig 4A shows *ex-vivo* permeation profiles from different BBVs compared to drug solution. For the determination of the free drug permeated through the intestine, as methanol is capable to disrupt the vesicle wall releasing the encapsulated drug, the drug was quantified directly in the samples withdrawn from the receptor compartment either without dilution with methanol or after dilution with methanol then injected into HPLC for the determination of the total drug permeated as free form and encapsulated drug permeated through transenterocytic transport. As illustrated in (Fig 4A) it is obvious that there is a significant difference between the permeation profiles of the different BBVs preparation through the intestine using non-diluted and methanol-diluted samples (p<0.05) confirming that most of the drug was transported in the encapsulated form. Drug solution showed the highest extent of permeation and there is no significant difference between the permeation profiles of the drug solution through the intestine using non-diluted and methanol diluted samples (p>0.05) as the drug was permeated in the free form. From (Fig 4B), it could be inferred that the ABBVs and Sito-GBBVs showed superior permeation than CBBVs up to 4 h of permeation. This could be due to the content of free DAC in each formulation system. The free drug is minimal in CBBVs as most DAC is encapsulated in the lipid bilayer of the vesicles and even the free DAC is adsorbed on the positively charged surface of the vesicles. The % of transenterocytic vesicular delivery of the ABBVs, CBBVs, Sito-GBBVs and drug solution are 84.1%, 93.1%, 85.7% and 0.4%, respectively. Although the CBBVs had the largest vesicular size in the examined BBVs, it showed superiority in transenterocytic vesicular delivery over other BBVs formulations. Positively charged BBVs facilitates electrostatic interaction with negatively charged cell membrane [48,49]. Positively charged nanocarriers permeability through gastrointestinal mucous barrier is easier than neutral and negatively charged ones due to the presence of negatively charged proteins in the external surface of gastrointestinal epithelial cells [50].

### *In-vitro* cytotoxicity and cellular uptake in HepG2 cell line

Cytotoxicity of different concentrations of the tested samples was tested using HepG2 cell line. The results were used to determine the IC_50_% values of each formulation of BBVs. So we can determine the appropriate concentration required for subsequent cellular uptake study. DAC solution exhibited the highest cytotoxicity against HepG2 with IC_50_% of 17.2μg*/*ml. This value increased to 22μg*/*ml for ABBVs and 25μg/ml for Sito-GBBVs. While DAC loaded CBBVs IC_50_% decreased to 19.7μg*/*ml.

In order to investigate the specific delivery of the ABBVs, CBBVs, Sito-GBBVs to liver cells compared to drug solution, the cellular uptake and internalization were tested in HepG2 cells. DAC internalization into HepG2 cells was evaluated quantitively by HPLC method and by fluorescence imaging. The cellular uptake study was performed to test the behavior of the different BBVs formulations in comparison to DAC solution after 8 h incubation with HepG2 cells. The cellular uptake of all BBVs formulation was significantly higher than that of DAC solution. It was found that the cumulative intracellular concentrations of the ABBVs, CBBVs, Sito-GBBVs and drug solution after 8 h post incubation were 18.2%, 19.1%, 19.6% and 5.2% respectively. Comparing these values, it was found that the cellular uptake of the BBVs formulations was about 3 folds greater than that of drug solution.

**Fig 5:**
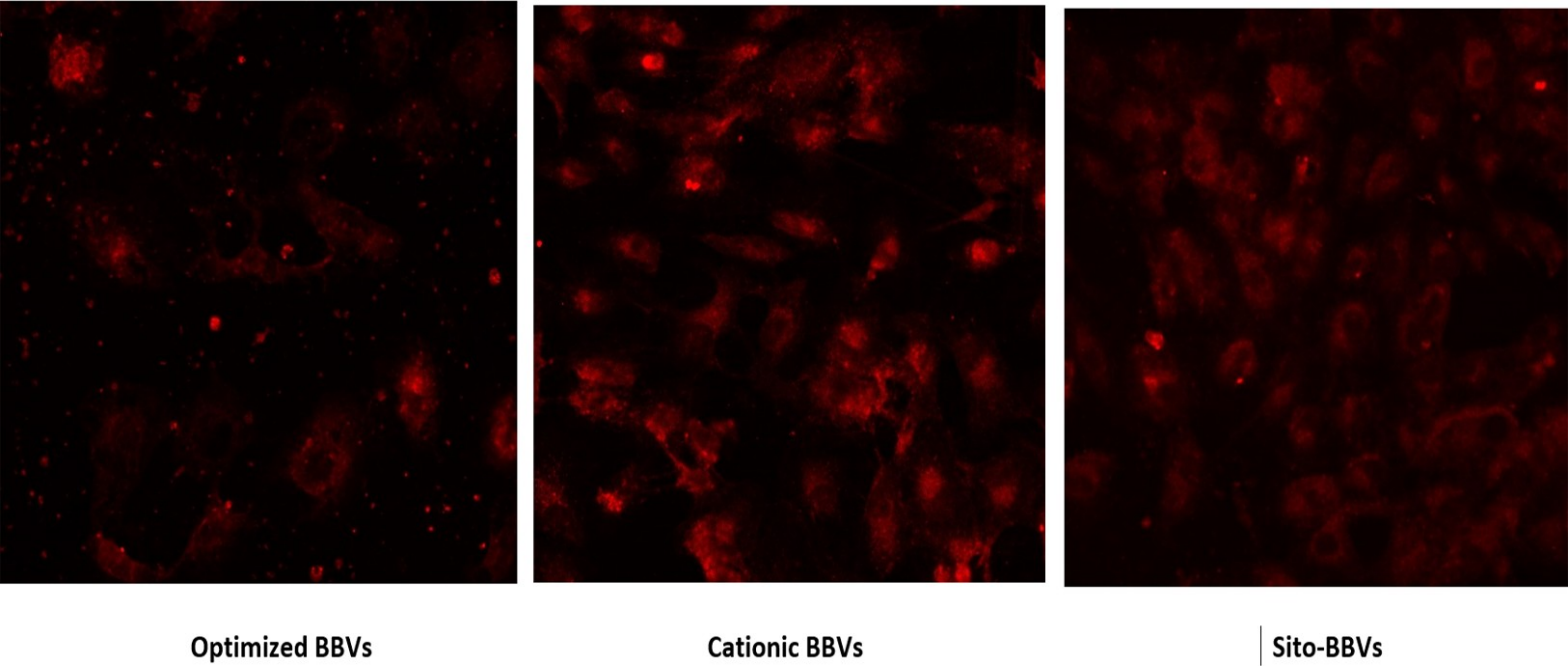
Confocal laser scanning microscopy (CLSM) images of HepG2 cells after incubation with Rh B labelled BBVs for 2 h at 37°C. (a) ABBVs, (b) CBBVs and (c) Sito-GBBVs.

To further investigate the internalization of the ABBVs, CBBVs and Sito-GBBVs into hepatocytes, the internalization of Rh B-loaded ABBVs, CBBVs and Sito-GBBVs after 2 h incubation with HepG2 cells was determined using fluorescence imaging by CLSM. The fluorescence intensity was determined using Software ZEN lite from ZEISS Microscopy. Rh B was chosen as a model lipid-soluble fluorophore to imitate the role of a lipophilic drug incorporated in BBVs [37,51]. As illustrated in (Fig 5), the CBBVs images showed highest fluorescence intensity (5899.2±1145.3) indicating the highest cell internalization. The fluorescence intensity of CBBVs was significantly higher than that of the ABBVs (2818.8±449.2) and Sito-GBBVs (4062.5±504.3), (p<0.05). The positively charged nanocarriers are broadly transported to hepatocytes due to interaction with anionic group of ASGPR binding site [23,25,26]. The internalization of Sito-GBBVs was facilitated by Clathrin-mediated endocytosis through interaction of Sito-G with ASGP-receptors on the hepatocytes cells. Sito-G containing BBVs showed significant higher fluorescence intensity than that of negatively charged BBVs (p<0.05).

### Protein adsorption resistance of the prepared BBVs in serum

**Fig 6:**
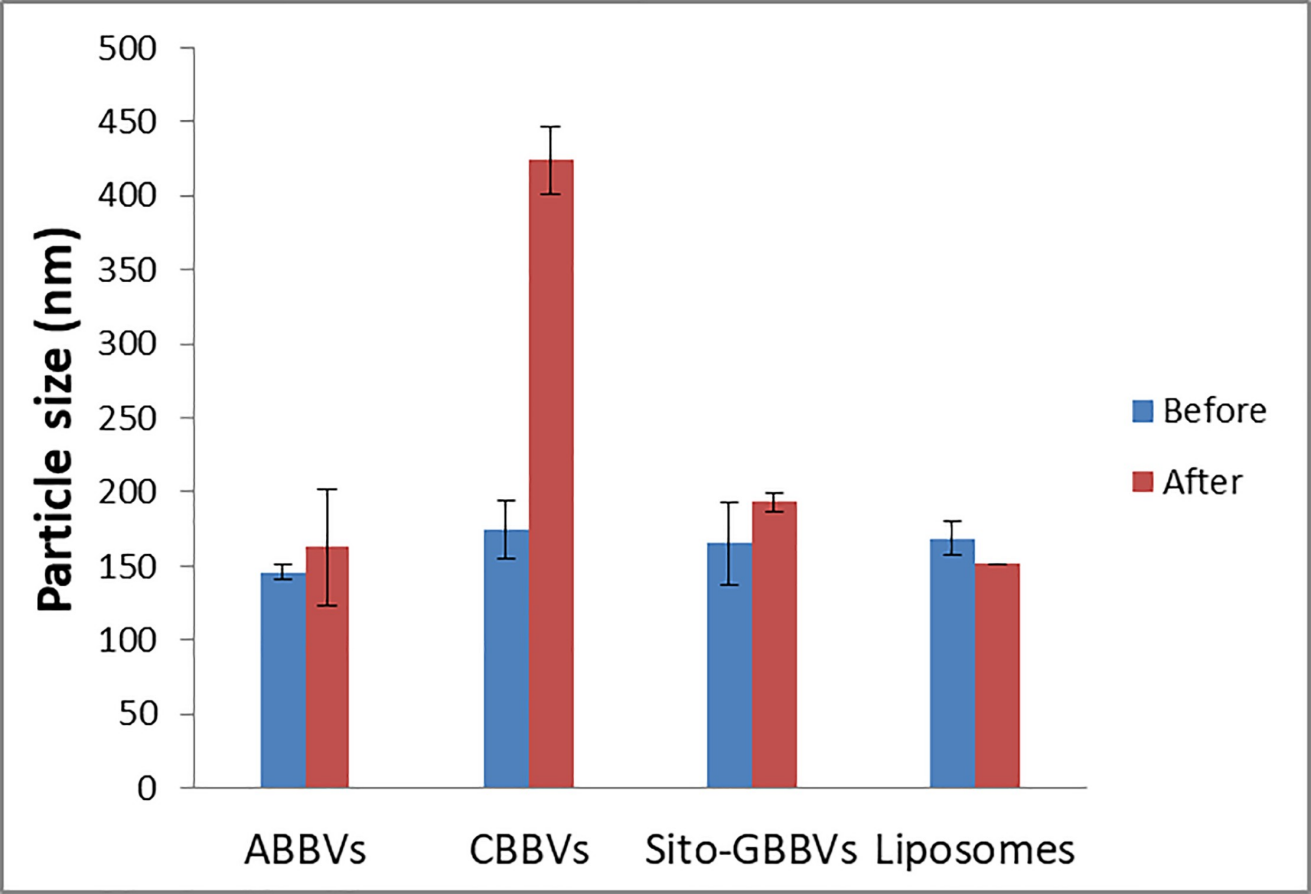
Representative bar chart of particle size of DAC loaded ABBVs, CBBVs, Sito-GBBVs and conventional liposomes before and after 2h incubation with rat serum.

For evaluation of BBVs stability against protein adsorption, the change in their particle size after incubation with serum was evaluated [52] as protein adsorption (Opsonization) onto BBVs makes them more noticeable to phagocytic cells [19]. This regularly facilitates instant clearance from the circulation system and affects their bio-distribution [53]. Fig 6 shows the particle size measured after incubation with rat serum for 2 h. The particle size of CBBVs increased significantly (p<0.05) indicating the adsorption of serum proteins on positive surfaces forming a ‘protein corona’ due to the negative nature of proteins [54]. Sito-GBBVs and ABBVs showed non-significant increases in particle size (p = 0.078 and p = 0.350, respectively) confirming the capability of both to resist adsorption of protein. Conventional liposomes showed a significant decrease of particle size (p = 0.023) which might be due to the low negative zeta potential of liposomes and the emulsifying properties of serum albumin during the dilution procedures.

## Conclusion

In this study, anionic, cationic and Sito-G decorated bile based vesicles (BBVs) were successfully prepared. The three formulations of BBVs showed acceptable stability in SGF and SIF compared to conventional liposomes due to incorporation of bile in the lipid wall of the vesicles. CBBVs showed superiority in transenterocytic transport through intestinal membrane as intact vesicles and HepG2 internalization (about 2.1 folds that of the ABBVs and 1.45 folds that of Sito-GBBVs) but suffered from opsonization in serum which would lead to rapid clearance by Kupffer cells. The optimum transenterocytic vesicular delivery, resistance to protein adsorption and HepG2 internalization propose that DAC loaded Sito-GBBVs administered orally may subsequently constitute an improvement in the treatment of hepatitis C infection. Further *in-vivo* studies are presently investigated.
